# An assessment of strategies to control dental caries in Aboriginal children living in rural and remote communities in New South Wales, Australia

**DOI:** 10.1186/s12903-018-0643-y

**Published:** 2018-10-29

**Authors:** Yvonne Dimitropoulos, Alexander Holden, Kylie Gwynne, Michelle Irving, Norma Binge, Anthony Blinkhorn

**Affiliations:** 1 Poche Centre for Indigenous Health, University of Sydney, Room 223 Edward Ford Building, Sydney, New South Wales Australia; 2 University of Sydney School of Dentistry, Mons Road, Westmead, Sydney, NSW 2145 Australia

**Keywords:** Aboriginal, Oral health promotion, Community

## Abstract

**Background:**

A community-led oral health service for Aboriginal people in Central Northern NSW identified the need for oral health promotion, as well as dental treatment; in three remote communities with limited access to dental services. A three-stage plan based on the Precede-Proceed model was used to develop a school-based preventive oral health program. The program will be piloted in three schools over 12 months aimed at improving the oral health of local Aboriginal children.

**Methods:**

The proposed program includes four components: daily in-school toothbrushing; distribution of free fluoride toothpaste and toothbrushes; in-school and community dental health education and the installation of refrigerated and chilled water fountains to supply a school water bottle program. Primary school children will be issued toothbrushing kits to be kept at school to facilitate daily brushing using a fluoride toothpaste under the supervision of trained teachers and/or Oral Health Aides. School children, parents and guardians will be issued free fluoride toothpaste and toothbrushes for home use at three-monthly intervals. Four dental health education sessions will be delivered to children at each school and parents/guardians at local community health centres over the 12 month pilot. Dental education will be delivered by an Oral Health Therapist and local Aboriginal Dental Assistant. The program will also facilitate the installation of refrigerated and filtered water fountain to ensure cold and filtered water is available at schools. A structured school water bottle program will encourage the consumption of water. A process evaluation will be undertaken to assess the efficiency, feasibility and effectiveness of the pilot program.

**Discussion:**

The proposed program includes four core evidence-based components which can be implemented in rural and remote schools with a high Aboriginal population. Based on the Precede-Proceed model, this program seeks to empower the local Aboriginal community to achieve improved oral health outcomes.

**Trial registration:**

TRN: ISRCTN16110292 Date of Registration: 20 June 2018.

## Background

Dental caries is a serious problem for Australian Aboriginal children [1]. For example, Aboriginal children living in New South Wales (NSW) experience on average 2.64 decayed, missing or filled teeth (dmft/DMFT) due to dental caries. This is near double the dmft/DMFT rate of 1.54, experienced by non-Aboriginal children in NSW [2]. When left untreated, dental caries can cause severe pain, and negatively impact a child’s quality of life, ability to concentrate at school, and capacity to eat, speak and socialize without embarrassment [3].

Improving the oral health of Aboriginal children is a priority in the Oral Health 2020: A Strategic Framework for Dental Health in NSW [4], and the NSW Aboriginal Oral Health Plan [5]. It is essential that strategies aimed at improving the oral health of Aboriginal children are sustainable, supported by the local Aboriginal community and culturally competent [6].

Culturally competent health promotion programs for Aboriginal people should be developed in consultation with Aboriginal communities and designed to meet the needs of specific communities [5]. Additionally, they should be culturally and linguistically appropriate, evidence-based, sustainable, implemented in collaboration with communities and evaluated [7]. In rural and remote communities, programs that solely rely on the input from dental professionals are often not sustainable given the general shortage of qualified professionals [4]. Therefore, prevention programs that can be delivered by the local Aboriginal community are likely to be more sustainable and suitable for the needs of that population.

In 2013, Aboriginal Elders in Central Northern NSW, identified three Aboriginal communities that had extremely limited access to dental services and needed preventive oral health care programs. Subsequently, the Poche Centre for Indigenous Health and the Centre for Oral Health Strategy were invited into these communities to work in partnership with the local Aboriginal community to introduce sustainable dental services and preventive oral health care programs for Aboriginal people in the region [8] In 2014, a community-led oral health service was established, which provides comprehensive dental treatment for Aboriginal people in Central Northern NSW. The service operates using clinicians who have relocated to the region and local Aboriginal people who have been trained and employed as dental assistants. Portable dental equipment is used to provide dental treatment to Aboriginal children and adults from schools and local community health centres. The local Aboriginal community still however, maintained the need for preventive oral health care programs to be implemented alongside dental treatment provided in schools and community health centres.

**Fig. 1:**
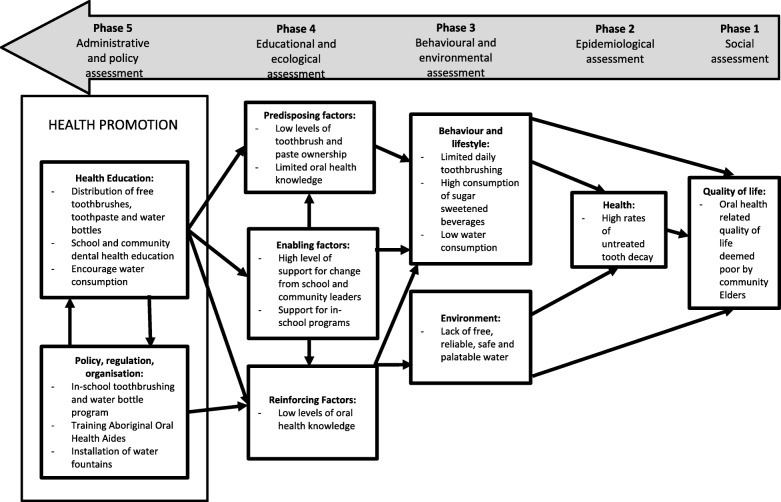
Application of Precede-Proceed model for health promotion planning to plan and implement oral health promotion in three rural and remote communities in NSW

In response to the community’s request for preventive oral health care programs, a three-stage plan based on the Precede-Proceed model of health program planning was used to develop a sustainable, community-led preventive oral health program (Fig. 1).

The Precede-Proceed model was used as it is a popular planning tool for population health programs [9]. It consists of a series of phases to assist researchers plan, design, implement and evaluate health programs. The first set of phases (PRECEDE) includes planned assessments to inform the design of the health program. The second set of phases (PROCEED) involves implementing a health program based on information learnt from the PRECEDE phases [10].

Guided by the Precede-Proceed model, Stage 1 included an epidemiological assessment of all Aboriginal children aged 5–12 years enrolled in local schools as well as an educational and ecological assessment of the community to determine predisposing risk factors and reinforcing and enabling factors to inform a targeted oral health program. The number of decayed, missing and filled teeth were recorded as well as baseline oral health knowledge and oral hygiene practices of children, parents/guardians, school staff and health workers.

The baseline data collected as part of Stage 1 data provided valuable planning information. The majority of children (87.5%) had untreated dental caries. The mean number of decayed primary teeth (dt) was 4.1 and the mean number of decayed permanent teeth (DT) was 0.7, indicating a clear need for dental treatment and education to prevent dental disease [11].

Ecological and educational assessment of the community identified four predisposing risk factors associated with an increased risk of developing dental caries:

1. Low levels of tooth brush ownership
2. Infrequent daily toothbrushing with a fluoride toothpaste
3. Frequent sugar consumption
4. High intake of sugar-sweetened beverages rather than drinking tap water

Based on the risk factors identified, the following oral health promotion strategies for Aboriginal children living in Central Northern NSW were developed, namely: increasing fluoride use through daily toothbrushing; ensuring safe and refreshing tap water is accessible to encourage the consumption of water rather sugar sweetened beverages; providing culturally competent oral health and nutrition education; and providing training programs to build capacity of the local Aboriginal community and existing health workforce to ensure oral health promotion is led and supported by the community.

The results of Stage 1 and proposed oral health promotion strategies were presented in a leaflet and also verbally reported back to the local Aboriginal people at an open community forum (locally known as a community ‘yarn up’) which is held to discuss local issues and events. The ‘yarn up’ included local Aboriginal Elders, teachers and school principals from the three local schools and representatives from the Poche Centre for Indigenous Health. The strategies were well-received at the ‘yarn up’ and members verbally agreed to the development of an oral health promotion program to be implemented in each of the schools in the three communities; which was later formalised in writing. This led to Stage 2 and thus the ‘implementation phase’ of the Precede-Proceed model.

Stage 2 involved developing a school-based oral health promotion program based on the findings of Stage 1. The program includes:

1. Daily in-school toothbrushing
2. Distribution of free fluoride toothpaste and toothbrushes to children and families
3. In-school and community dental health education
4. Installation of refrigerated and chilled water fountains to supply a school water bottle program

The four components are based on existing programs which have shown to be effective in other Aboriginal communities [6, 12], and utilising high quality systematic reviews [13]. This study protocol describes the components of the proposed school-based program and evaluation protocol to determine the feasibility, efficiency and effectiveness of the strategies to control dental caries in Aboriginal children living in rural and remote communities in NSW.

The aim of the program is to improve the oral health of Aboriginal children by promoting daily toothbrushing using fluoride toothpaste, increasing oral health knowledge and encouraging the consumption of water to reduce the reliance on sugar-sweetened beverages.

Stage 3 will pilot the proposed program for 12 months in three schools in Central Northern NSW that enroll a high proportion of Aboriginal children. The process evaluation to be undertaken at the completion of the pilot, is based on the Precede-Proceed model for process evaluation (Phase 6) and will determine the program’s feasibility, efficiency, effectiveness and overall satisfaction of the participating communities.

## Methods

### Daily in-school toothbrushing with a fluoride toothpaste

Daily in-school toothbrushing will be implemented in all Kindergarten to Year 6 (primary) classes in the three schools.

At the commencement of the pilot, all children enrolled in Kindergarten to Year 6 will be issued with a toothbrushing kit. This kit will be labeled for each child and kept at school for daily toothbrushing. It includes a toothbrush, 1000 ppm fluoride toothpaste and hard plastic storage case for the toothbrush and paste.

Children will brush their teeth at school once per school day under the supervision of a trained teacher and/or Oral Health Aide. The program will allow the school to choose which combination will work at their school, whether this will be the existing classroom teacher, the introduction of an Oral Health Aide or a combination of the two. This approach will be used as the program has been designed to be led and implemented by the local community. The Oral Health Aide must be a local Aboriginal person to promote a culturally safe environment for children to brush their teeth at school, this may include providing individual children with support to brush their teeth. They will be employed by the school for 1 h per day and will assist the classroom teachers to implement the program by supervising toothbrushing.. The role of an Oral Health Aide was incorporated into the design of this study as a significant barrier to in-school toothbrushing programs is the demands on a teacher’s time [6]. It is based on the Children’s Oral Health Initiative (COHI) in Canada which utilizes ‘COHI Aides’ to support oral health promotion activities and provide oral health education [14]. Teachers and Oral Health Aides will receive a NSW TAFE skillset in Infection Control (HLTIN30IC) and First Aid (HLTAID003) to ensure they can supervise in-school toothbrushing safely.

All labeled toothbrush cases will be stored in a ventilated carrier next to the sink in each classroom and a replacement toothbrushing kit will be issued to each child every three months which will coincide with the commencement of each new school term.

The following outcomes will be used during the process evaluation to measure the reach and efficiency of daily in-school toothbrushing;

- At least 70% of Aboriginal children enrolled in the school have consented to participate in the program
- 90% of children with consent to participate will brush their teeth at least 170 out of the 200 school days
- 80% of children with consent to participate are continuing to brush their teeth each day at school after six months
- Teacher support for the program both at the time of commencement and six months later is positive.

### Distribution of free fluoride toothpaste and toothbrushes to communities

A strategic approach will be used in this program to distribute free fluoride toothpaste and toothbrushes to communities on a three-monthly basis to encourage the social norm of toothbrushing.

This approach will include:

- All children who participate in daily in-school toothbrushing will receive a second toothbrushing kit every three months to take home for home use
- Senior school children (Years 7–12) will receive a toothbrushing kit every three months to take home for home use
- Parents/guardians at three-monthly intervals will receive free 1000 ppm fluoride toothpaste and toothbrushes every three months during dental health education sessions.

### In-school and community dental health education

Dental health education sessions will be delivered to school children and parents and guardians on a three-monthly basis over the pilot period to encourage the uptake of oral hygiene resources which will be distributed.

At the commencement of each new school term (three-monthly basis), a half-hour dental health education session will be delivered to children at each school. Therefore, a total of four sessions will be delivered at each school over the pilot period. Topics covered will be: encouraging brushing twice daily with fluoride toothpaste, restricting sugar intake and encouraging the consumption of water.

Parents/guardians will receive dental health education sessions at the local community health centre every three months (total of four sessions over 12 months). Topics will be: encouraging brushing twice a day with a fluoride toothpaste, demonstrating toothbrushing using a mouth model, restricting sugar intake and encouraging the consumption of water and information on seeking regular dental care. All education sessions will be delivered by an Oral Health Therapist and a local Aboriginal dental assistant.

Dental health knowledge and oral hygiene practices of children and parents/guardians will be collected following the completion of the pilot using the interviewer-assisted questionnaires which were used to collect baseline data in Stage 1. Results will be compared to baseline data to assess the impact of dental health education and the provision of free oral toothbrushes and toothpaste.

The following outcomes will be used during the process evaluation to measure the impact of dental health education and the provision of free oral toothbrushes and toothpaste:

- 90% of Aboriginal children have a toothbrush and toothpaste at home
- 90% of Aboriginal children brushed their teeth in the last 24 h
- 70% of parents and/or guardians are satisfied with the toothbrushing program
- 70% of parents and/or guardians assist their children to brush their teeth each night
- A 70% increase in dental health knowledge specifically relating to the importance of the primary dentition, prevention of tooth decay and the effects of giving a baby a bottle of milk to bed.

### School water bottle program

The school water bottle program will ensure filtered and refrigerated water is available on school grounds to reduce the reliance on sugar-sweetened beverages brought into schools. As part of the program, each school will be offered refrigerated and filtered water fountains or refrigeration and filtration units installed to existing fountains depending on the school infrastructure. The water fountains will not remove fluoride nor provide additional fluoride to the water supply. They have been incorporated into this program to provide children with a safe, refreshing and free alternative to sugar-sweetened beverages, which are known to increase a child’s risk in developing dental caries. Parents/guardians will be informed of the availability of refrigerated and filtered water at school and education regarding the effects of sugar-sweetened beverages on teeth will be delivered to school canteen staff. To encourage the consumption of water, each child will receive a free drinking water bottle to be kept at school and stored in a ventilated caddy in each classroom. Teachers will be instructed to ask children to fill their water bottle up each morning and encourage them to drink from their water bottles regularly throughout the day. Water bottles will be refilled three times per day to ensure they remain chilled.

The following outcomes will be used during the process evaluation to measure the impact and effectiveness of refrigerated and filtered water fountains:

- 70% of children drink water from the fountain every day
- 90% of children drink tap water daily
- 70% reduction of consumption of sugar-sweetened beverages on a daily basis in children aged 5–12 years
- 70% of teacher support the water fountain and water bottle program

### Data collection

As part of the process evaluation (Table 1), qualitative and quantitative data will be used to evaluate the efficiency, feasibility and effectiveness of the program. A focus group will be conducted with teachers and/or Oral Health Aides who directly supervise the toothbrushing program at each school. Therefore, a total of three focus groups will be conducted. A series of pre-determined questions relating to the implementation of the program, issues encountered, and perceived benefits will be used to guide the focus group. Focus groups will be recorded and transcribed verbatim. Thematic analysis will be used to determine themes pertinent to the implementation of school based toothbrushing programs looking at the attitudes and perceptions of teachers and oral health aides to the involvement of the schools in the programs.

**Table 2:**
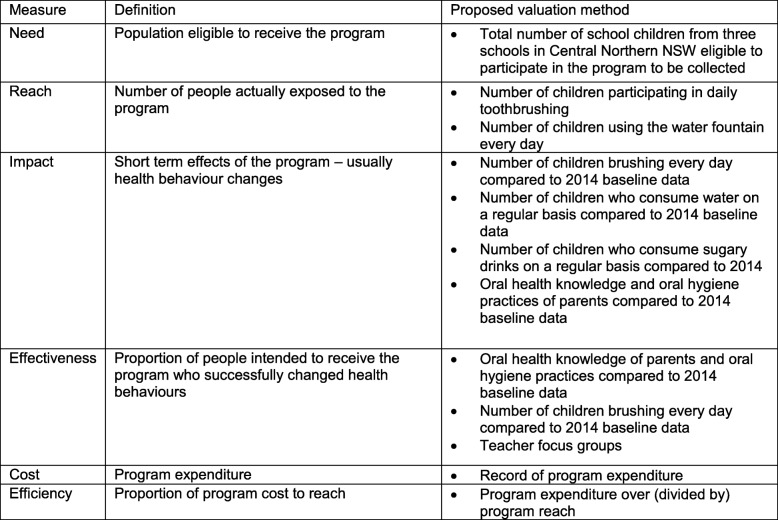
Process evaluation plan for proposed oral health promotion program based on Precede-Proceed health model process evaluation

A daily register will be used by each school to record student participation in the brushing program. Descriptive analysis of the data will be undertaken to calculate the proportion of children who brush their teeth daily.

The use of the water fountain by the children will be recorded by a researcher on two randomly chosen days over the 12 month pilot. Teachers and students will not the purpose of the researcher’s visit to minimise the posibility of altered behaviour. A record of maintenance issues for water fountains installed will also be kept to determine if the fountains are efficient.

An interviewer-assisted questionnaire will be undertaken at the completion of the pilot program with children aged 5–12 years to determine attitudes towards the fountain and changes in oral health knowledge and diet. An interviewer-assisted questionnaire will also be conducted at the completion of the pilot program with parents/guardians to determine changes in oral health knowledge. Questionnaire responses will be analysed using descriptive statistics (SPSS software, version 22 [SPSS Inc., Chicago Ill, USA]).

## Discussion

Dental caries is recognised as a preventable disease [15], however, Aboriginal children continue to experience higher levels of dental caries than their non-Aboriginal peers [2]. ‘Closing the gap’ in Aboriginal oral health has been identified as a State and National priority [5, 16]. Developing and implementing evidence based and sustainable oral health promotion programs has been identified as an important strategy to ‘Close the gap’ [5, 17]. The shortages of dental professionals in rural and remote communities only reinforces the need for programs to be developed that can be delivered by the local community.

The proposed program includes four evidence-based strategies which have been well documented in international literature to prevent and reduce dental caries. While some of these strategies have been implemented and evaluated in Australian Aboriginal communities [6], Aboriginal children continue to experience near double the rate of dental caries than non-Aboriginal children. Therefore, research into the effective implementation of these strategies that can control dental caries in Aboriginal communities (such as this study) is still needed.

The incorporation of fluoride into preventive oral health care programs is fundamental to ensuring programs are effective. Children who brush their teeth with fluoride toothpaste at least once daily are less likely to develop dental caries. This program proposes daily in-school toothbrushing using fluoride toothpaste for primary school students in Kindergarten to Year 6 which is to be supervised by a classroom teacher and/or Oral Health Aide. School toothbrushing programs often face staffing barriers and problems with infection control [6]. This program proposes two unique points of difference to overcome these barriers. Local Aboriginal Oral Health Aides will assist teachers supervise toothbrushing and teachers and Oral Health Aides will be offered a NSW TAFE skillset in Infection Control and First Aid to ensure they can supervise toothbrushing competently and mitigate infection control issues.

The utilisation of a local Aboriginal Oral Health Aide to supervise toothbrushing is based on the COHI in Canada which uses ‘COHI Aides’. The ‘COHI Aides’ are Indigenous people who have been specifically trained as oral health professionals, providing dental screenings, oral health education and apply fluoride varnish to improve the oral health of Indigenous children in Canada [14]. Aboriginal Oral Health Aides in time may become a recognised role for Aboriginal people in communities with limited access to oral health services to support the local implementation of community oral health promotion. The role of an Aboriginal Oral Health Aide could potentially include supporting local implementation of oral health promotion programs, delivering dental health education and applying fluoride varnish, similar to the role of ‘COHI Aides’ in Canada.

Additionally, this program includes a strategic approach to the distribution of free toothpaste and toothbrushes for home use every 3 months to all school students and parent/guardians. This aims to increase accessibility to oral hygiene implements in Aboriginal communities and encourage the social norm of toothbrushing at home.

Preventive oral health programs for Aboriginal children are more likely to be effective if they include dental health education for the wider community [6, 18]. Therefore, this program will provide three-monthly dental health education sessions for students in schools and parents/guardians at local community health centres. The dental health education delivered through this program aims to improve the oral health literacy of Aboriginal children and adults and encourage the uptake of oral hygiene implements which are to be distributed. -. The program proposes that dental health education is delivered by an Oral Health Therapist, accompanied by a local Aboriginal dental assistant to ensure the cultural competence of information given.

The frequent consumption of sugar-sweetened beverages, is associated with increased risk of developing dental caries [19].This program facilitates the installation of refrigerated and filtered water fountains in schools to ensure access to cold and filtered water at schools and reduce reliance on sugar-sweetened beverages. A structured water bottle program component which will be supervised by teachers aims to encourage the consumption of water, rather than sugar-sweetened beverages.

Genuine collaboration has been undertaken with the local Aboriginal community to ensure that these evidence-based strategies are implemented culturally competently, can be led and implemented locally and are sustainable. Oral health promotion in Aboriginal communities is often unsuccessful or ends prematurely due to unsustainable sources of funding or relying on only one individual in the community [6]. This program provides schools and communities the opportunity to work together to implement a suite of evidence-based strategies which are known to prevent and reduce dental caries in an innovative way in Aboriginal communities. This study protocol describes the implementation of four evidence based strategies in schools to improve the oral health of Aboriginal children in Central Northern NSW and may provide insight into the effective implementation of sustainable oral health promotion to reduce dental caries in Aboriginal children in NSW and Australia.These strategies include daily in-school toothbrushing, distribution of free fluoride toothpaste and toothbrushes, a school water bottle program and in-school and community dental health education.

## Abbreviations

ACCHS: Aboriginal Community Controlled Health Services
COHI: Children’s Oral Health Initiative
dmft/DMFT: Decayed, missing or filled teeth
NSW: New South Wales
